# Supplementary material for: Are carbon emissions trading and green financial instruments synergistic? -Comprehensive quantitative research based on content analysis
Source: PLoS One. 2024 Mar 7;19(3):e0298601. doi: 10.1371/journal.pone.0298601 (PMC10919601; doi:10.1371/journal.pone.0298601)
Supplement: S1 Data — (ZIP) [file pone.0298601.s001.zip › Data/地级市Z_F工作报告.zip!/╜¡╬≈/╛░╡┬╒≥╩╨/└┤╫╘╣1⁄2╓┌║┼í╛┬φ┐╦╩2╛▌═°í┐_2019.docx]

各位代表：

现在，我代表市人民政府向大会报告工作，请予审议，并请市政协委员和其他列席人员提出意见。

一、2018年工作回顾

2018年是全面贯彻党的十九大精神的开局之年，也是景德镇市经济社会发展极不平凡的一年。在省委、省政府和市委的坚强领导下，我们深入学习贯彻习近平新时代中国特色社会主义思想和党的十九大精神，从更高层次贯彻落实习近平总书记对江西工作的重要要求，坚定不移实施“三个五”战略行动，较好地完成了市十五届人大三次会议确定的目标任务，全市经济社会保持了健康发展的良好势头。

——经济运行总体平稳。生产总值增长8%左右；财政总收入132.5亿元，增长7.5%；一般公共预算收入90亿元，增长3.6%；规模以上工业增加值增长8.3%；固定资产投资增长11.2%；社会消费品零售总额340.7亿元，增长10.8%；外贸出口66亿元，增长20%；单位生产总值能耗下降3.2%。

——特色产业逐步壮大。三次产业结构达到6.5：49.5：44; 陶瓷及文化创意产业规上增加值增长8.5%；直升机及通航产业增加值增长18%；旅游总收入增长29.3%，旅游接待总人次增长23.5%。我市因“老工业基地调整改造力度较大，支持传统产业改造、培育新产业新业态新模式等工作成效突出”受到国务院通报表扬。

——城市品牌更加凸显。国务院同意创建景德镇国家陶瓷文化传承创新试验区，成为全国首个文化类国家级试验区。“中欧城市实验室”落户景德镇，成为中欧可持续城镇化合作首个项目。成功承办全国“双修”现场会暨试点经验交流会，“双修”试点工作得到住建部充分肯定并向全国推广。活化陶瓷工业遗存，加快推进新旧动能转换的典型经验做法受到国办通报表彰。乐平市土地节约集约利用工作获国务院表彰。

——环境质量持续改善。全年空气优良率96.4%，全省第一；PM2.5平均浓度31微克/m3，下降22.5%，PM10平均浓度56微克/m3，下降16.4%，空气质量在全省率先进入国家二级标准。地表水Ⅰ-Ⅲ类水质断面比例93.9%，饮用水各断面水质保持稳定，水质优良率100%。

——民生事业不断发展。10件民生实事基本完成。全年民生支出159.4亿元，增长9.8%；城镇企业职工基本养老保险参保人数25万人，完成年度任务102.3%；城镇新增就业2.9万人，完成年度任务125.4%；城镇居民可支配收入增长8.5%；农村居民可支配收入增长9%。

一年来，我们主要做了以下工作：

（一）注重培育产业，在转型升级中提升经济发展质量。加快推进陶瓷产业转型。组建了景德镇陶瓷集团；陶瓷智造工坊一期、红叶新厂基本建成；红叶陶瓷亮相平昌冬奥会，并首获美国巴拿马太平洋万国博览会金奖；澐知味公司荣获“中国质量奖提名奖”；中润陶瓷亮相亚运会；名镇天下等企业亮相首届中国自主品牌博览会。加快推进航空小镇建设。昌飞、德利、江直等整机项目进展顺利；19家航空零部件企业基本建成；昌飞成功申报江西省通航应急救援基地，赣翔通航成为全省首个具备直升机飞行驾驶私商照培训资质单位；新增两家科技协同创新体和一个海智工作站。加快推进汽车产业发展。依托北汽集团，加大昌河新产品研发力度，研发中心、发动机、新能源电池项目积极推进，零部件产业园加快建设。加快推进旅游资源整合。初步完成御窑厂遗址、浮梁古县衙创5A级景区规划编制；新增2个4A级、7个3A级景区；“陶溪川号”观光巴士旅游公交线路正式开通；花千谷、洪岩等重点旅游项目进展顺利；浮梁县获评全省旅游产业发展先进县；高岭？瑶里景区入选全省十大避暑目的地。同时，传统产业和新经济蓬勃发展。焦化集团更名黑猫集团，实现产业转型大跨越；华意压缩机获批工信部智能制造试点项目；天新药业进入江西民营企业百强前60名；乐平工业园被认定为省新型工业化创业基地；猎豹移动人工智能项目签约落户。

（二）注重城市建设，在“双创双修”中提升城市品质。以规划为引领，以问题为导向，围绕“一带两城十大行动”，扎实推进生态修复、城市修补。城市规划全域覆盖。完成河东老城区等控规、海绵城市等专项规划以及彭家弄等6个省级历史文化街区保护规划编制；省级城市设计试点有序开展；全市控规覆盖率达到95%，基本实现全覆盖。生态修复成效明显。累计完成山体修复53万平米，新增城市绿地287万平米，改造了松涛公园、森林公园，新建了如意湖公园，打造了浮梁段生态风光带、宝石码头、南河蓄洪湖公园等特色景观。城市功能日趋完善。地下综合管廊被评为2018年度中国管廊建设十大示范项目,在住建部、财政部联合开展的中央财政支持地下综合管廊试点城市2018年绩效评价中，我市在第二批15个管廊试点城市中获排名第2的好成绩；城区第一条隧道——凤凰山隧道竣工通车；打通“断头路”20余条，新建、改建、扩建城市道路34条；打造了30个“15分钟便民圈”，完成263条背街小巷改造，规划建设了一批农贸市场、停车场、垃圾中转站、公厕、街心公园和中小学校。同时，我们还成功入选全国文明城市提名城市，顺利完成创建第六届全国文明城市的首年测评工作。

（三）注重创新引领，在改革开放中提升城市竞争力。深入实施“放管服”改革。按照“一窗受理”“一次不跑” 或“只跑一次”的要求，进一步优化行政审批事项，行政权力事项减少2549项，精简率达到79.6%。持续开展“降成本优环境”专项行动，累计为企业减负26亿元；完善工业项目“容缺受理”等制度，提升项目落地效率；企业注册开办时限降至5个工作日，新增登记注册企业28977户，增长41.2%。加快推进投融资体制改革。国控集团、陶文旅集团、城开投公司资产总额达到1200亿元左右。创新城市管理体制机制。“公安+城管”、“消防+环卫”等新模式成效显现，“环卫一体化”改革向乡镇延伸，园林市场化顺利推进。统筹推进其他各项改革。以珠山区马鞍岭为试点的农业农村“三个三”改革，探索出一条激活集体经济、带动农民致富的新路；机构改革、昌南新区设立以及国企改革等稳步推进。开放活力显著增强。依托赣深、赣京、赣港会等平台，大力实施招大引强工程，全年利用外资增长9.1%，第15届瓷博会创造了境外参展企业、参观游客等多项新高；加快推进招才引智工程，出台人才引进“十条政策”，人才落地条件进一步放宽；加强校地合作，与清华大学签订横向合作协议，构建合作办学新模式；加大科研投入，高新技术企业总数增长80%，成为全省第二个全社会研发投入强度突破2%的设区市；加强对外通达能力建设，开通了景德镇至莫斯科中欧班列，航班覆盖全国12个大中城市，皖赣铁路改线工程建成并通车，昌景黄高铁正式开工。

（四）注重文化传承，在对话世界中提升发展软实力。文化保护工作稳步实施。御窑厂遗址数字化展示工程有序推进；御窑博物馆完成主体工程；陶溪川老厂房入选第三批中国20世纪建筑遗产项目名录、第一批中国工业遗产保护名录；联合故宫博物院等申报古陶瓷修复研究国家文物局重点科研基地；连续三年举办御窑厂出土瓷器与故宫博物院文物对比展；《御窑厂遗址保护管理条例》正式实施，《高岭土古矿遗址保护规划》获国家文物局批准。乐平市被中国民间文艺家协会命名为“中国古戏台之乡”，古戏台营造技艺入选“第一批国家传统工业振兴目录”。文创产业蓬勃发展。陶溪川文创街区获批海峡两岸青年就业创业基地、省首批大众创业万众创新示范基地；国际陶瓷文创小镇入选省第二批特色小镇创建名单；新增3家省级文化产业示范基地。扩大文化交流成效显著。举办了改革开放40周年景漂景归文化艺术展，在南非举办了“感知中国？丝路瓷行”中国陶瓷文化展，在德国举办了“遇见中国—纪念马克思诞辰200周年系列文化展”、在荷兰举办了“故宫瓷器—皇帝御用”暨“景德镇御窑陶瓷特展”，陶瓷文化影响力、城市知名度显著提升。

（五）注重环境保护，在环保整改中提升生态环境质量。扎实推进国家生态文明试验区建设，全力实施“净空、净水、净土”三大行动，严格落实“河长”、“湖长”、“林长”等制度。开展蓝天保卫战，严厉管控烟花爆竹燃放，大力整治扬尘、燃煤锅炉等污染。加大环保设施建设，有机废弃物处理厂投入运行，投入20亿元完善了城市污水管网、改造了老南河截污干管、推进了污水处理厂扩容提标工作。建立土壤污染防治协调工作机制，开展了乐安河流域重金属污染农田土壤治理和重点危废专项排查。全力配合中央环保督察组开展“回头看”工作，及时解决环境整治面临的新问题，污染防治攻坚战取得阶段性成果。乐平共库列入全国和全省水源地重点保护区，洪岩国家森林公园申报获专家评审通过。浮梁县被评为第二批国家生态文明建设示范县和 首批“绿水青山就是金山银山” 省级实践创新基地。

（六）注重“三农”工作，在乡村振兴中提升城乡融合水平。坚持农业农村优先发展，稳步实施乡村振兴战略。粮食总产量达到66.9万吨，再创历史新高；“高岭·中国村”田园综合体、浮梁荻湾乡村振兴开发及乐平磻溪河田园综合体等项目进展顺利；乐平市入选2018年创建“全国农村一二三产业融合发展先导区”和全国县域经济强县；浮梁县被评为2018全国茶叶百强县；全市农业产业化龙头企业达到144家，销售收入增长9%。全力打好脱贫攻坚战，有序推进产业扶贫“四个一”工程，机关干部挂点帮扶实现全覆盖，完成4190名贫困人口和5个省级贫困村退出任务,启动城镇贫困群众脱贫解困工作。以“一带三边五线”为重点，累计投入资金8.7亿元推进1040个新农村点建设和农村人居环境整治，乡村环境向整洁干净宜居转变。

（七）注重以人为本，在共享发展中提升群众生活品质。加大民生投入，解决了一批群众关心事、烦心事和身边事。新政务服务中心搬迁启用；景德镇学院搬迁工程加快实施，九所中小学（幼儿园）建设进展顺利，教育资源配置更趋合理。公立医院综合改革继续深化，基层卫生计生服务体系进一步健全。全面完成11个体育场馆的新建和改扩建，成功举办了一届极具特色、极为出色的省运会，开闭幕式展演精彩纷呈，我市体育健儿创历史最好成绩，金牌总数和团体总分均列全省第二。建成日供水15万吨的新洋湖水厂（一期），有效保障了东城区用水安全；快速推进浯溪口水利枢纽工程库区建设和移民安置工作；积极解决中心城区房地产开发项目历史遗留问题，认真做好 6户困难集体企业改制和8户供销系统企业职工安置工作。加强社会治安防控，创新完善全警情处置管控机制，深入推进扫黑除恶专项斗争，全面推进阳光信访、责任信访、法制信访，群众安全感进一步提升。安全生产态势平稳，是全省唯一连续16年未发生重大事故的设区市。同时，国防动员、拥军优抚、人民防空、防震减灾等工作进一步加强，民族宗教、档案史志、机关事务管理、统计、外侨、工青妇、科协、文联、社联、残联、红十字、老龄等各项工作都取得新成效。

一年来，我们按照市委统一部署，落实全面从严治党要求，积极开展忠诚型、创新型、担当型、服务型、过硬型政府建设，大力倡导“事事马上办、人人钉钉子、个个敢担当”的精神，坚决纠正“怕、慢、假、庸、散”等作风顽疾，工作作风有了新改进，行政效能有了新提升。同时，我们坚持依法行政，认真落实市人大及其常委会决定，自觉接受人大、政协监督，及时办理人大代表议案建议83件、政协委员提案164件。加强财政资金使用管理，强化审计监督，加强廉政建设，进一步营造了风清气正政务环境。

各位代表！过去一年的成绩来之不易，这是市委科学决策、坚强领导的结果，是全市人民团结一心、砥砺奋进的结果！在此，我代表市政府，向全市人民，向人大代表和政协委员，向各民主党派、工商联、无党派人士、各人民团体和社会各界人士，向驻景部队官兵和省属各单位，向关心支持景德镇发展的海内外朋友，表示衷心的感谢并致以崇高的敬意！

同时，我们也清醒地看到，我市发展不平衡不充分的问题仍然突出，政府工作还存在一些不足和挑战。主要有：受外部环境影响，经济下行压力很大，国民生产总值增速低于年初预定目标0.5个百分点左右；经济总量不大、主导产业不强仍然存在；中心城区集聚辐射带动能力还有待提升；金融服务实体经济能力还有待提高；优质公共服务供给离群众需求还有一定差距；政府部门办事效率、担当意识、服务水平还有待进一步提高，等等。我们一定高度重视这些问题，采取更有力的措施认真加以解决。

二、2019年工作安排

2019年是新中国成立70周年，是全面建成小康社会关键之年，做好各项工作至关重要。今年政府工作的总体要求是：高举习近平新时代中国特色社会主义思想伟大旗帜，全面贯彻党的十九大、十九届二中、三中全会和中央经济工作会议精神，认真落实省委十四届六次、七次全会和市委十一届五次、六次全会精神，以“双创双修”为主线，以创建国家陶瓷文化传承创新试验区为抓手，以“十大工程”为重点，全力实施“三个五”战略行动，着力推进“五型”政府建设，为打造与世界对话的国际瓷都接续奋斗，以优异成绩庆祝新中国成立70周年。

今年全市经济社会发展的主要预期目标是：生产总值增长8%—8.5%；财政总收入增长5.5%左右；规模以上工业增加值增长8.5%左右；固定资产投资增长10.5%左右；社会消费品零售总额增长10.5%左右；实际利用外资增长6%左右；城镇居民人均可支配收入增长8%左右；农村居民人均可支配收入增长8.5%左右；居民消费价格总水平涨幅控制在2.9%左右；节能减排完成省下达任务。

要实现上述目标，必须抢抓国家发展的重要战略机遇期，在落实“巩固、增强、提升、畅通”八字方针上下功夫，突出项目建设，推动高质量跨越式发展。重点抓好八个方面工作：

（一）全力实施“十大工程”，让国际瓷都更具魅力。今年，我们将重点实施古镇重塑、新区发展、陶瓷复兴、工业壮大、旅游名城、乡村振兴、引智留才、生态美丽、城市文明、民生保障“十大工程”，全力推进341个重大项目，把国际瓷都建设目标化、项目化、责任化。

千方百计争引项目。立足城市特色和产业优势，加快建设一批强基础、管长远、利大局的重大项目库。紧紧围绕中央和省里政策导向，找准上级政策、产业布局和资金投向的切入点、契合点、着力点，争取更多项目纳入上级盘子。整合各类资源优势，深入开展“三请三回”和“三企”入景行动，争取更多项目落户。大力开展招商引资，围绕“3+1+X”产业实施产业链招商、市场化招商、精准招商，引进10亿元以上项目不少于12个，其中国家级高新区引进50亿元以上项目至少1个，省级园区引进20亿元以上项目至少1个。

全力以赴建设项目。突出老城保护，大力实施以御窑博物馆为核心、以“三陶”为重点的古镇重塑工程，着力建设最具特色、最有文化底蕴的千年古镇。突出产业发展，以园区为平台，加快推进以航空小镇、汽车小镇、陶瓷小镇为重点的产业项目，以项目提质增效促进产业集群化、高端化发展。突出人才支撑，大力实施引智留才工程，全力推进景德镇学院搬迁及配套、景德镇陶瓷大学扩建等12个项目，为引进人才、留住人才提供保障。

持之以恒落实项目。严格落实工程建设项目审批制度，建立项目审批“一窗受理”、“绿色通道”，精简优化审批流程，缩短项目审批时限，着力提高项目开工率和竣工率。严格落实市、县领导联系制度，重点项目每月调度，重大项目随时调度，滞后项目密集调度，对10亿元以上重大项目、亿元以上重点项目，分层建立项目推进工作专班，提供全程协调服务，促使项目尽快落地开工、竣工投产。多渠道保障项目资金，加快盘活闲置资源，优化低效资产，努力向政策、向市场、向资源要资金；支持投融资平台壮大资产规模、提高信用等级，力争国控、陶文旅信用评级达到AA+，拓宽融资渠道，降低融资成本。

（二）全力推进“双创双修”，让生态家园更加美丽。以“双创双修”为主线，按照“精心规划、精致建设、精细管理、精美呈现”的要求，大力实施治脏、治乱、治堵、功能修补、生态修复、特色彰显、亮化美化、治理提升“八大行动”，全面提升城市功能与品质。

着力提升城市风貌。充分发挥规划设计的引领作用，围绕展现地域风情、时代风貌、人文风格，加快全市控规和专项规划编制工作，力争控规覆盖率达100%；启动昌南新区、青塘片区等城市设计，完善老城区修规设计，编制老城业态功能规划；实施“中欧共建城市实验室”项目，制定中欧文化城市指标体系，打造中欧文化城市会客厅，启动数字博物馆和规划信息平台建设。继续实施一批“治山理水、显山露水”项目，开展珠山大桥—中渡口码头历史风貌恢复、景北大桥—宝积寺景观提升工程，打造白鹭大桥—岚山大桥景观带，启动瓷都大桥—宝石码头慢行系统建设，加快景南等片区景观修复，启动凤凰山及云门公园、昌南片区等生态提升项目，开展绿化、美化、彩化、珍贵化造林工程，着力彰显山水园林特色。

着力完善城市功能。重点实施十大城市功能项目：推进站前四路、建设北大道、光明大道延伸段、凰鸣路、古窑片区等道路建设及通站路整治、4个交叉口道路渠化工程；加快老南河综合改造及部分片区防洪排涝设施建设；启动官庄片区防洪堤坝工程、下窑泵站及拦水闸项目；启动东、西汽车客运站和铁路货运东站搬迁工作，新建鱼山货运码头，提前布局铁空水陆多式联运；引进2-3个商贸综合体；改造提升部分老旧小区、背街小巷及城中村；新建停车场、垃圾中转站等公建设施；推进科技馆、城市规划馆、青少年宫项目建设；启动大型农贸批发市场、物流园二期和货运分装场建设，推进枫树山农贸市场、昌江区农贸市场建设；加快推进新区医院及中小学校等项目建设，不断完善城市功能布局。

着力提升管理水平。进一步深化城管体制改革，巩固环卫一体化、园林市场化、“公安+城管”“消防+环卫”成果。强化街道、社区公共服务职能，规范提升小区物业管理。利用大数据创新城市管理，推进“互联网+”便民服务，加快建设智慧城市。全面推行网格化管理，保持打击“两违”高压态势。持续开展交通秩序、经营秩序等专项整治，营造良好城市秩序。加快推动烟花爆竹禁燃、历史文化名城保护、地下管廊（管网）管理等立法工作。继续开展“健步走、美生活、护平安”活动，最大限度汇聚城市建设管理和“双创”工作正能量。继续加大创建全国文明城市和国家卫生城市工作力度，挂图作战，对标创建，把创文创卫落到实处。

打好污染防治攻坚战。严格执行生态保护红线，统筹山水林田湖草系统治理，全面推进国家生态文明试验区建设。紧盯环保督察反馈问题整改，突出抓好城市污水、饮用水源、工业固废等环境治理。打好蓝天、碧水、净土、长江经济带“共抓大保护”等攻坚战，严格落实河长制、湖长制、林长制，集中力量解决扬尘污染、土壤污染、医疗废物污染等突出问题。加快构建政府主导、企业主体、社会组织和公众参与的环境治理体系，努力形成生态环境保护大格局。

（三）全力推进试验区建设，让陶瓷文化更加繁荣。牢牢把握国家陶瓷文化传承创新试验区建设的战略机遇，推进国家陶瓷文化保护传承基地、国家陶瓷产业创新发展基地、世界陶瓷人才集聚高地、世界著名陶瓷文化旅游目的地、国际陶瓷博览交易中心、国际陶瓷文化交流合作中心建设，着力擦亮千年瓷都“金字招牌”。

推进陶瓷文化传承创新发展。着力打造国家陶瓷文化保护传承基地。深入挖掘保护陶瓷遗址、遗迹、遗产，以御窑厂为核心，陶阳里、陶溪川、陶源谷为重点，全面推进古窑址、历史文化街区、工业遗产保护利用；加快实施御窑厂大遗址本体保护、周边提升、数字化展示等项目，推进御窑厂遗址申报世界文化遗产；完成御窑博物馆内部展陈并对外开放；启动陶阳里历史街区保护利用、老城区近现代陶瓷工业遗产综合保护及刘家弄、葡萄架等历史街区重点要素保护项目；加大非物质文化遗产保护传承力度，推动景德镇手工制瓷技术申报世界非物质文化遗产，延续城市历史文脉。着力打造国家陶瓷产业创新发展基地。围绕千亿级陶瓷产业集群，大力实施陶瓷龙头企业培育工程，优化陶瓷产业发展布局，推动手工制瓷精品化、日用陶瓷规模化、科技陶瓷高端化；围绕陶瓷全产业链，打通从原料供应、工业设计、制造加工、检测鉴定到营销物流等各个环节；加快推进“国际陶瓷文创小镇”、“陶大小镇”、洛客设计谷、陶青台文创园等项目建设，打造陶瓷产业创新发展新地标。

搭建陶瓷文化交流合作平台。着力打造世界陶瓷人才集聚高地。以推进景德镇陶瓷大学“双一流”建设工程为契机，推进产学研合作，着力培养陶瓷后备人才；加大陶瓷人才引进力度，推动陶瓷相关领域国家重大人才工程人选和项目落户，支持院士工作站、博士后科研流动站建设。着力打造国际陶瓷文化交流合作中心。借助全球创意城市网络、国际友城等平台，广泛开展国际陶瓷学术研修、文化交流，促进陶瓷文化“走出去”；进一步提升瓷博会办展水平，加快瓷博会国际化、专业化、市场化步伐；精心承办第十三届全国美术作品展览陶艺展区活动。

建设陶瓷文化对外展示窗口。着力打造世界著名陶瓷文化旅游目的地。大力实施“陶瓷+”行动，推动陶瓷与文化、旅游等各领域跨界融合发展，培育陶瓷文化旅游核心产品。积极整合旅游资源，发展文化旅游新业态，打造层级分明、功能互补的文化旅游体系。着力打造国际陶瓷博览交易中心。加快建设景德镇陶瓷（国际）博览平台和陶瓷产业大数据中心，全力帮助陶瓷企业打响品牌、保护知识产权、拓展对外贸易，抢占高端陶瓷市场。积极运用大数据平台，规划建设陶瓷电商园。规范陶瓷服务业及陶瓷产业链市场，大力引进国家级外贸综合服务试点企业落户，建设景德镇陶瓷垂直电商平台，打造陶瓷电商集聚区和电商孵化基地。

全面实施陶瓷复兴工程。围绕陶瓷全产业链，重点推进十大陶瓷产业项目：红叶新基地形成年产高档日用瓷、陈设瓷、艺术瓷1000万件生产能力；邑山瓷业形成年产2000万件生产能力；开工建设年产20万吨陶瓷坯釉原料的陶瓷原材料生产基地；整合资源，建立全市陶瓷产品检测中心；完成总投资20亿元的智造工坊二期主体工程；开工建设古作坊群；开工建设“窑坞印象”大型实景演出项目；开工建设总投资20亿元的陶瓷产业孵化器；完成唐英创意设计园主体工程；力争引进3—5家高科技陶瓷企业。

（四）全力推进军民融合，让航空发展更加坚实。抢抓军民融合国家战略，按照“厂所品牌、政府平台、市场机制”原则，以航空小镇建设为载体，全力做大做强航空产业。

全面推进航空项目建设。重点推进十大项目：加快吕蒙总装园二期建设，确保AC313、AC310、S300等整机项目建成投产，力争实现年产直升机和无人机300架以上；加快推进德利直升机设计制造项目，实现年产150架直升机能力；加快推进江直JH系列直升机批产，力争年产200架直升机；推动总投资20亿元公务机整机制造项目落户；以昌飞、602所采购订单方向为主线，围绕航电、旋翼、复合材料等关键领域，推动园内20余家企业建成投产；加快昌飞通航机场建设；启动集航空游乐、航空演艺、科技展示等功能一体的航空主题乐园项目前期；开工建设航空体验中心；建成优飞智能无人机项目并投产；力争引进一批零部件配套企业、直升机关联企业及通航企业。

加快推进产城融合发展。大力推进国家通航产业综合示范区建设，积极申报创建国家军民融合创新示范区。坚持多规合一，高标准规划建设航空小镇。优化提升交通路网，加快推进一批道路新建、改造、提升工程。大力实施主次干道污水管网、企业环境提升改造等治理项目。进一步夯实平台支撑，加快推进航空科技园一期项目，完成昌南？慧谷航空产业孵化中心建设，力争新增标准化厂房33万平方米以上。充分发挥政府购买通航服务等政策引领作用，大力发展航空+运动、航空+旅游、航空+科普等新业态，不断丰富通航产业内涵。

着力打造航空产业集群。充分发挥602所、昌飞的技术引领作用，推动航空中小企业集聚创新、爆发式增长，不断提升企业核心竞争力和产业引领力，努力形成航空龙头企业强、配套企业多、产业集群实力雄厚的新局面。积极争取重型直升机落户，切实提高民机生产份额，强化零部件配套能力。支持昌飞开工建设吕蒙工程中心大楼、公寓及吕蒙厂区基础设施，加快飞行服务站和民机科研试飞基地建设。支持602所完成直升机研发平台拓展建设。支持江直加快推进航空科技馆项目。支持上海和利、华夏九州、江西赣翔等通航企业拓展飞行员培训、应急救援、农林植保等服务。

（五）全力推进创新驱动，让转型发展更具活力。围绕推动高质量发展的要求，深入实施创新驱动发展战略，加快推进传统产业转型升级、新兴产业快速成长。

着力强化科技创新支撑。深化与大厂大所大学的战略合作，搭建科技创新发展平台，力争新增一批省级以上众创空间、重点实验室（工程技术中心）等平台载体。完善以企业为主体、市场为导向、产学研用中介金融相结合的技术创新体系，支持驻景科研院所科技成果就地转化，加快培育一批科技创新型企业。推进智能制造产业发展，促进互联网、大数据、人工智能与制造业深度融合。加大招才引智力度，积极对接国家“千人计划”专家团队，加快培育本土人才，柔性引进“高精尖缺”人才，吸引更多优秀人才在景创新创业。支持各类创新工厂、创客空间、虚拟创新创业社区发展。有序推进国家03专项推广应用示范，促进移动物联网应用及产业发展。继续做好鄱阳湖自主创新示范区创建工作。

着力挖掘产业发展动能。重点实施十大产业提升项目：深化与北汽集团战略合作，推进北汽昌河研发中心建设，大力发展新能源汽车产业；支持黑猫集团 “走出去”，延伸发展新材料、环保、文旅等三产服务业；鼓励制冷压缩机产品升级换代，推动华意高效变频压缩机年内竣工投产；推进江西美菱环保节能冰箱扩产项目年内竣工；支持天新、宏柏、金龙、世龙、富祥等企业增资扩产，打造化工细分行业领军企业；加快推进猎豹移动人工智能制造项目；引进物流、会展、咨询、信息等中介与专业服务业项目，推动传统服务业提档升级；引进工业设计、大数据、服务外包等高技术服务业；加快建设电子商务中心，努力打造完善的电子商务产业生态圈；大力发展金融服务业，推进一批金融证券机构落户,深入实施企业上市“映山红”行动，推动“个转企、小升规、规改股、股上市”。

着力强化园区平台建设。深入实施开发区改革和创新发展三年攻坚行动计划，探索建立异地项目建设利益共享机制，优化产业发展布局，力争高新区主营业务收入达800亿元，陶瓷工业园区、乐平工业园区主营业务收入达500亿元。按照“工业成本、景区风貌、城市功能”要求，大力实施园区功能提升计划，不断完善园区路网、管网等设施建设，科学布局学校、医院、超市、酒店、金融等功能配套。坚持“以亩产论英雄”导向，积极推进土地集约节约利用，积极开展扩园工作，为产业发展预留空间。

（六）全力推进旅游发展，让城市名片更具特色。整合生态资源、文化资源和城市品牌资源，加快创建国家全域旅游示范区，推动我市由旅游资源大市向旅游经济强市转变。

深度挖掘旅游市场。依托“六山两湖”和六大世界遗产中心的区位优势，借助赣东北旅游联盟等纽带，积极参与国家和省级旅游推介等活动，大力宣传景德镇旅游品牌。创新营销手段，精心办好中国旅游日、茶文化节等节庆活动，大力发展旅游商品，挖掘瓷都美食文化，不断丰富旅游产业内涵。

着力提升旅游环境。大力发展智慧旅游，推进旅游线上营销平台、大数据中心、旅游集散中心等建设，完善全域旅游道路标识系统，健全旅游服务配套设施。加强景区景点管理，推进瑶里等景区体制机制创新，巩固提升4A景区资质。强化旅游行业管理，积极引进知名旅行社，大力扶持本土旅行社，切实提高旅游地接能力。发展制瓷演艺、自驾车营地等新业态，加快建设落地自驾游服务体系。

加快建设旅游精品。坚持旅游与生态、文化深度融合，重点实施十大旅游项目。以御窑厂遗址创5A级景区为目标，积极推进“景德古镇”项目；以陶溪川创5A级景区为目标，推进陶溪川二期建设；开展浮梁古县衙5A级景区创建工作；以三宝瓷谷创4A级景区为目标，建设三宝国际瓷谷旅游提升项目；以名坊园创4A级景区为目标，建设一批餐饮、演艺、购物等配套项目；引进新建1-2家高端酒店，培育一批经济型酒店、精品主题民宿等层次分明、类型多样的住宿产品；推进洪岩旅游特色小镇等生态旅游风景区竣工；打造以陶瓷、航空为主题的十大研学游基地；推进凤凰小镇、宋瓷小镇等项目落地；启动市游客集散中心建设。

（七）全力推进乡村振兴，让城乡发展更加协调。围绕农业稳粮优供增效、农村整洁美丽和谐宜居、农民脱贫致富文明，全面实施乡村振兴战略，促进城乡融合互动、一体化发展。

深化农业农村改革。完善农村基本经营制度，落实农村承包地“三权分置”政策，鼓励发展多种形式适度规模经营；继续深化农村土地、集体产权、集体林权和农田水利等改革，着力培育壮大村级集体经济；稳妥推进“两权”抵押贷款试点，促进生产要素向农业农村倾斜；逐步推广农业农村“三个三”改革经验，推动农村更美、农民更富。

改善农村人居环境。大力推进中心集镇建设，不断完善集镇功能。围绕“一带三边五线”，深入开展农村环境整治，重点建设1000个新农村点，打造景瑶线、乐弋线、206国道沿线等美丽风景示范线。加快推进农村生活垃圾治理、农村厕所革命，推动农村人居环境与乡风文明双提升。

培育壮大特色农业。大力发展品牌农业、绿色农业、规模农业，推进“乐平菜、浮梁茶”等地域品牌规模化、产业化，力争实现蔬菜播面 60万亩、产量150万吨、产值30亿元，茶园总面积25万亩、总产量2万吨、总产值突破20亿元目标。实施三产融合攻坚计划，着力打造以陶瓷文化、古村落文化、乡村生态旅游为特色产业的样板村，促进农业与文化、生态融合发展。深入推进高标准农田建设，确保粮食播面130万亩，产量稳定在55万吨。加快现代农业发展，支持乐平市生态农业科技园区申报创建景德镇国家农业科技园区。

推进农业项目建设。重点实施十大农业项目：加快推进48万亩高标准农田、高岭？中国村田园综合体、浮梁荻湾乡村振兴开发、花千谷农都小镇、浮梁茶10万亩扩改提升、乐平磻溪河田园综合体、花正红农业科技园、魁杨联圩除险加固等项目，开工建设农产品直批电商物流博览园和万亩名贵树木改造项目。

统筹城乡融合发展。围绕提高中心城市辐射带动力和首位度，建设功能互补、资源共享、互联互通的“半小时经济圈”。重点改造升级G351国道、G206国道浮梁蛟潭—景德镇—乐平段、湘官公路，推进“四好农村路”建设和危桥改造工作，增加城乡公交线路，不断拉近中心城市与乐平、浮梁的时空距离。加快昌景黄高铁建设，推进机场迁建项目，打通部分城际道路，提升区位优势。统筹市域公共服务设施布局，促进城乡人口、资源要素双向流动。

（八）全力推进社会事业，让群众生活更加美好。始终坚持财力、政策、资源“三倾斜”，做到民生投入只增不减，为老百姓办实事只增不减，群众获得感、幸福感、安全感只增不减。

倾力办好民生实事。今年，重点办好十件实事：完成8000户城市棚户区改造；提供2300个公益性岗位，帮助困难群体就业，发放创业贷款4.2亿元，扩大景漂创业人员信誉担保范围；购置90辆纯电动公交车，新建公交候车亭100座，新增3条城区公交线路；升级改造公路145公里、城市道路18公里；新建、改建城乡公厕160座，完成农户改厕1.4万户；新建10个城乡居家养老服务中心（站），新建一个孤儿区域性养育中心；新建55所标准化卫生计生服务室，实现全市行政村全覆盖；建设10条全民健身路径，满足群众休闲健身需求；启动中心城区2所学校建设；新建占地1000余亩的殡仪馆和公墓区。

坚决打好脱贫攻坚战。围绕“两不愁三保障”目标，因地制宜，精准施策，确保4个省级贫困村摘帽、6384人脱贫。把短期“退得出”和 长期“不返贫”相结合，进一步健全扶贫机制，落实扶贫政策，推进产业扶贫“四个一”工程，实现“1+5”模式全覆盖，不断提高脱贫质量。扎实做好城镇贫困群众脱贫解困工作，确保全面小康路上一个都不少。

加快发展民生社会事业。大力发展教育事业，积极引进国内一流学校开展合作办学；加快推进学校在建工程，确保景德镇学院新校区和9所在建中小学（幼儿园）秋季开学交付使用。进一步深化公立医院综合改革，加快推进部分医院改扩建项目，全面落实10种大病免费救治和15种大病专项救治政策。完善公共文化服务体系，推进公共文化服务设施建设，支持景德镇中国陶瓷博物馆申报国家一级博物馆，加强乐平古戏台文化保护利用。大力发展体育事业，广泛开展全民健身运动，以成功承办省运会为契机，加强体育场馆综合利用，争取承办更多国家级赛事，促进群众体育和竞技体育协调发展。把稳就业摆在突出位置，进一步落实大众创业、万众创新各项政策，重点解决好高校毕业生、农民工、退役军人等群体就业。推进社会保险费征收体制改革，确保社保费现有征收政策稳定。

创建和谐平安瓷都。加强和创新社会治理，有效预防和化解社会矛盾。深入开展扫黑除恶专项斗争，严厉打击各类违法犯罪活动。牢牢守住安全生产基本盘，确保不发生重大及以上事故。紧盯“一个上升、两个下降、三个提高”工作目标，加强信访工作，依法及时就地解决群众合理诉求。继续巩固双拥创建成果，做好退役军人服务保障工作。

同时，积极支持工青妇、红十字会等群团组织发挥更大作用，扎实做好统计、外侨、人防、对台、宗教、档案、气象、水文、防震减灾等各项工作。

三、加强政府自身建设

打造与世界对话的国际瓷都，对政府自身建设提出了更高要求。我们将聚焦“怕、慢、假、庸、散”突出问题，提振精气神，围绕提升政府施政能力和服务水平，扎实推进“五型”政府建设，进一步强本领、转作风、优环境、促发展。

第一，坚持忠诚干事的政治本色。牢固树立“四个意识”，切实增强“四个自信”，自觉践行“两个维护”，在政治立场、政治方向、政治原则、政治道路上同党中央保持高度一致。始终坚持把党对一切工作的领导贯彻到政府工作的各个领域、各个方面，确保中央大政方针和省委省政府、市委的决策部署落地生根。

第二，崇尚敢为人先的首创精神。广泛学习借鉴先进地区的好经验、好做法，坚决破除封闭僵化保守思维，倡导运用新理念、新办法推动政府工作。稳步推进机构改革，加快构建职责明晰、运行高效的政府治理体系。深化“放管服”改革，加强政府规范性文件的合法审查、后续保留和及时清理，深入推进“互联网＋政务服务”，打造政务服务“一网通办”品牌，努力实现县级政务服务“一门服务、一窗受理”，加快打造“四最”营商环境。

第三，弘扬敢于担当的实干之风。加快政府职能转变，制定明晰权责清单，不断提高政府工作人员整体素质和担当意识。坚决为想干事、能干事、干成事者撑腰鼓劲，坚决克服有风险不敢干、有难度不愿干、没有批示不办事、不见纪要不执行等不作为现象。深入开展重点工作大督查，健全督查考核问责机制，着力解决一批历史遗留问题，坚决整治不担当、不作为、懒政怠政行为。创新政府管理方式，推广数字化综合办公系统，加强绩效考核管理，建立并落实奖惩机制，全面提升政府工作效率。

第四，强化依法行政的法治意识。始终坚持依法行政，做到“法无授权不可为，法定职责必须为”，以法治思维和法治方式用好“有形之手”、管住“妄动之举”。切实加强行政与司法协调联动，健全公示听证、决策咨询、专家论证等制度，不断提高政府决策科学化、民主化、法治化水平。健全政务公开机制，认真办好《政府公报》，不断增强政府工作透明度。加强政务诚信建设，进一步提升政府公信力。自觉接受人大、政协、监察、群众及舆论等监督。

第五，恪守清正廉洁的为政底线。进一步强化全心全意为人民服务的宗旨意识，为群众诚心诚意办实事、尽心尽力解难事、坚持不懈做好事。牢固树立过紧日子思想，强化绩效预算评估，提高资金使用效益。严格贯彻落实中央八项规定精神，坚决杜绝“四风”问题隐形变异、反弹回潮。坚持把纪律和规矩挺在前面，带头履行“一岗双责”，带头执行自律准则，不过“防线”、不踩“红线”、不越“底线”，推动全面从严治党不断向纵深发展。

各位代表！实干托举梦想，奋斗铸就辉煌！让我们更加紧密地团结在以习近平同志为核心的党中央周围，高举习近平新时代中国特色社会主义思想伟大旗帜，从更高层次贯彻落实总书记对江西工作的重要要求，在省委、省政府和市委坚强领导下，以思想的再解放、改革的再攻坚、行动的再落实，全力实施“三个五”战略行动，为努力打造与世界对话的国际瓷都接续奋斗，共同谱写新时代江西物华天宝、人杰地灵新画卷的景德镇篇章，以优异成绩庆祝新中国成立70周年！
